# Biosynthesis of copper oxide nanoparticles mediated *Annona muricata* as cytotoxic and apoptosis inducer factor in breast cancer cell lines

**DOI:** 10.1038/s41598-022-20360-y

**Published:** 2022-09-28

**Authors:** Rana I. Mahmood, Afraa Ali Kadhim, Sumayah Ibraheem, Salim Albukhaty, Harraa S. Mohammed-Salih, Ruaa H. Abbas, Majid S. Jabir, Mustafa K. A. Mohammed, Uday M. Nayef, Faizah A. AlMalki, Ghassan M. Sulaiman, Hassan Al-Karagoly

**Affiliations:** 1 Department of Biomedical Engineering, College of Engineering, Al-Nahrain University, Baghdad, Iraq; 2 Department of Biology, College of Science, Mustansiriyah University, Baghdad, Iraq; 3 Al_kindy College of Medicine, University of Baghdad, Baghdad, Iraq; 4 Department of Chemistry, College of Science, University of Misan, Maysan, 62001 Iraq; 5 College of Medicine, University of Warith Al-Anbiyaa, Karbala, Iraq; 6 Department of Orthodontics, College of Dentistry, University of Baghdad, Baghdad, 10011 Iraq; 7 College of Dentistry, Al-Farahidi University, Baghdad, Iraq; 8 Department of Applied Sciences, University of Technology, Baghdad, 10066 Iraq; 9 Department of Medical Physics, Al-Mustaqbal University College, Babylon, 51001 Iraq; 10 Department of Biology, College of Science, Taif University, P.O. Box 11099, Taif, 21944 Saudi Arabia; 11 Department of Internal and Preventive Medicine, College of Veterinary Medicine, University of Al-Qadisiyah, Al Diwaniyah, 58002 Iraq

**Keywords:** Cancer, Molecular biology, Nanoscience and technology

## Abstract

This study investigated for the first time a simple bio-synthesis approach for the synthesis of copper oxide nanoparticles (CuO NPs) using *Annona muricata L* (*A. muricata*) plant extract to test their anti-cancer effects. The presence of CuONPs was confirmed by UV–visible spectroscopy, Scanning electron microscope (SEM), and Transmission electron microscope (TEM). The antiproliferative properties of the synthesized nanoparticles were evaluated against (AMJ-13), (MCF-7) breast cancer cell lines, and the human breast epithelial cell line (HBL-100) as healthy cells. This study indicates that CuONPs reduced cell proliferation for AMJ-13 and MCF-7. HBL-100 cells were not significantly inhibited for several concentration levels or test periods. The outcomes suggest that the prepared copper oxide nanoparticles acted against the growth of specific cell lines observed in breast cancer. It was observed that cancer cells had minor colony creation after 24 h sustained CuONPs exposure using (IC_50_) concentration for AMJ-13 was (17.04 µg mL^−1^). While for MCF-7 cells was (18.92 µg mL^−1^). It indicates the uptake of CuONPs by cancer cells, triggering apoptosis. Moreover, treatment with CuONPs enhanced Lactate dehydrogenase (LDH) production, probably caused by cell membrane damage, creating leaks comprising cellular substances like lactate dehydrogenase. Hence, research results suggested that the synthesized CuONPs precipitated anti-proliferative effects by triggering cell death through apoptosis.

## Introduction

In the past several years, the domain of material science has extensively focused on synthesizing nanoparticles with changeable morphology and defined dimensions, shape, and order. Much research has been conducted concerning nanoparticles and their immense applications for biosensing and chemo, energy conserving devices, drug delivery mechanisms, catalysis and electrochemistry, optoelectronics, optics, and medicinal therapeutics antibacterial, anticancer, and antimicrobial agents^1–4^. Nanoscale size, distinct shape, and extensive surface area are advantages for their marked catalytic characteristics. Using post-functionalization techniques, additional surface changes for the materials create distinctive physical characteristics^5^. Numerous physical and chemical methods are being assessed for synthesizing NPs using bottom-up or top-down approaches^6^. However, a majority of synthetic techniques require severe conditions with high temperature and pressure and potent reducing substances, producing nefarious by-products *in-situ,* which are discouraged by green protocols^7,8^. Several scenarios use cost-intensive and challenging techniques. Hence, the demand for eco-friendly nanotechnology is increasing to facilitate the benefits of clean, biometric, and ecological processes for nanoparticle production^9,10^.

Biological techniques for synthesizing NPs using plant leaf extracts or organisms like fungi, bacteria, and algae are considered eco-friendly substitutes for chemical synthesis because such approaches are nontoxic and less cost- and energy-intensive^11^. Plant extract-based NP production approaches are more beneficial than environmentally friendly biological approaches because cell cultures are not required^12^. Moreover, NP production using plants is beneficial because of safe handling and easy availability associated with plants and their extensive metabolite content to facilitating reduction^13^.

MCF-7 is an adherent, epithelial cell regarded One of the most important contributions of the MCF-7 cell line to breast cancer research has been its utility for the study of the estrogen receptor (ER) alpha, as this cell line is one of a very few to express substantial levels of ER mimicking the majority of invasive human breast cancers that express ER^14^. Plants contain many phytochemicals and synthesize several secondary metabolites that are potentially usable for Cu and CuO NPs production. Flavonoids and phenols are among the vital phytochemicals present in different plant parts like roots, leaves, flowers, shoots, stems, and fruits. Such phenolic substances contain ketone and hydroxyl groups, facilitating iron chelation and possessing potent antioxidant characteristics^15,16^. NPs produced using this green approach enhance stability, reduce deformation and aggregation of NPs, and facilitate phytochemical adsorption from NP surface, contributing to higher reaction speed^17^. Research works suggest that plant-based Cu/CuO NPs exhibit antitumor properties for colon^18^, breast^18^, blood (leukemia)^18^, liver^19^, cervical^20^, ovarian, skin (epithelioma)^21^, lung^21^, and gastric cancers^22^. *Annona muricata L.* (*A. muricata*) from the Annonaceae family has been extensively researched in the last few decades because of its therapeutic benefits. The use of Annonaceae family plants for medicinal benefits has been suggested long back; consequently, these species have been researched extensively because of their traditional uses and potent bioactivity^23,24^. Medicinal plants are regarded as essential for healthcare benefits globally. Chronic degenerative conditions have assumed epidemic proportions; these are severe health concerns, causing treatment options to be given due clinical attention^25^. Data about specific cytotoxic characteristics of *A. muricata* reported ethnobotanically has raised the species’ popularity as an antitumor agent^26^. In-vitro studies indicated that extracts affected cancerous cells more immensely than normal cells; most extracts did not cause cytotoxicity for healthy human cells, indicating selectivity^27^. Experiments indicated that *A. muricata* leaf-based hydroalcoholic extracts with 1.6 mg/mL and 50 mg/mL concentrations enhanced non-tumor cell viability, while 100 mg/mL did not change viability^28^. Reports suggest such selectivity speeds healing without many side effects. The present study was conducted by employing *A. muricata* as a reducing agent to synthesize CuO nanoparticles and assess potential therapeutic anticancer activity.

## Results and discussion

### Characterizing the synthesised CuONPs

**Figure 1:**
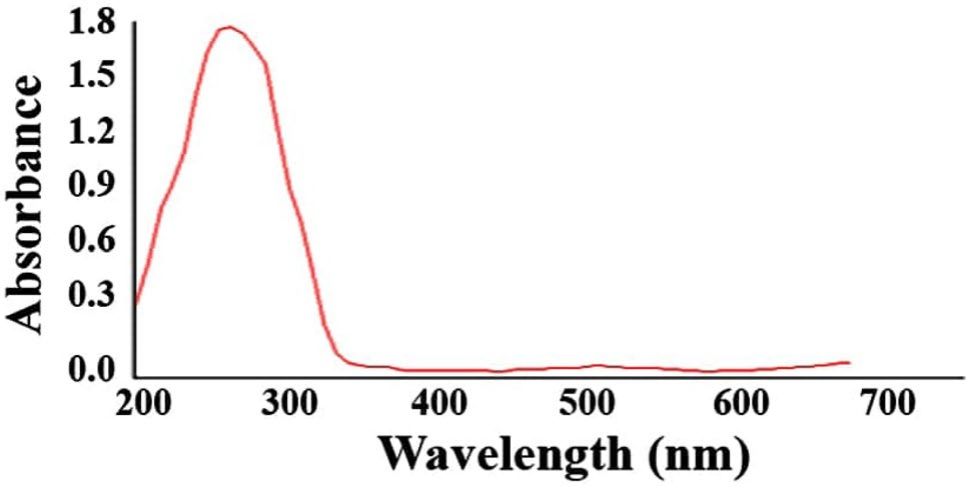
UV-spectroscopy of CuONPs.

Exposure to *A. muricata* peel extract was used to prepare CuO NPs, assessed using UV spectroscopy. As depicted in Fig. 1, the prepared CuONPs peaked around 260 nm^29,30^.

**Figure 2:**
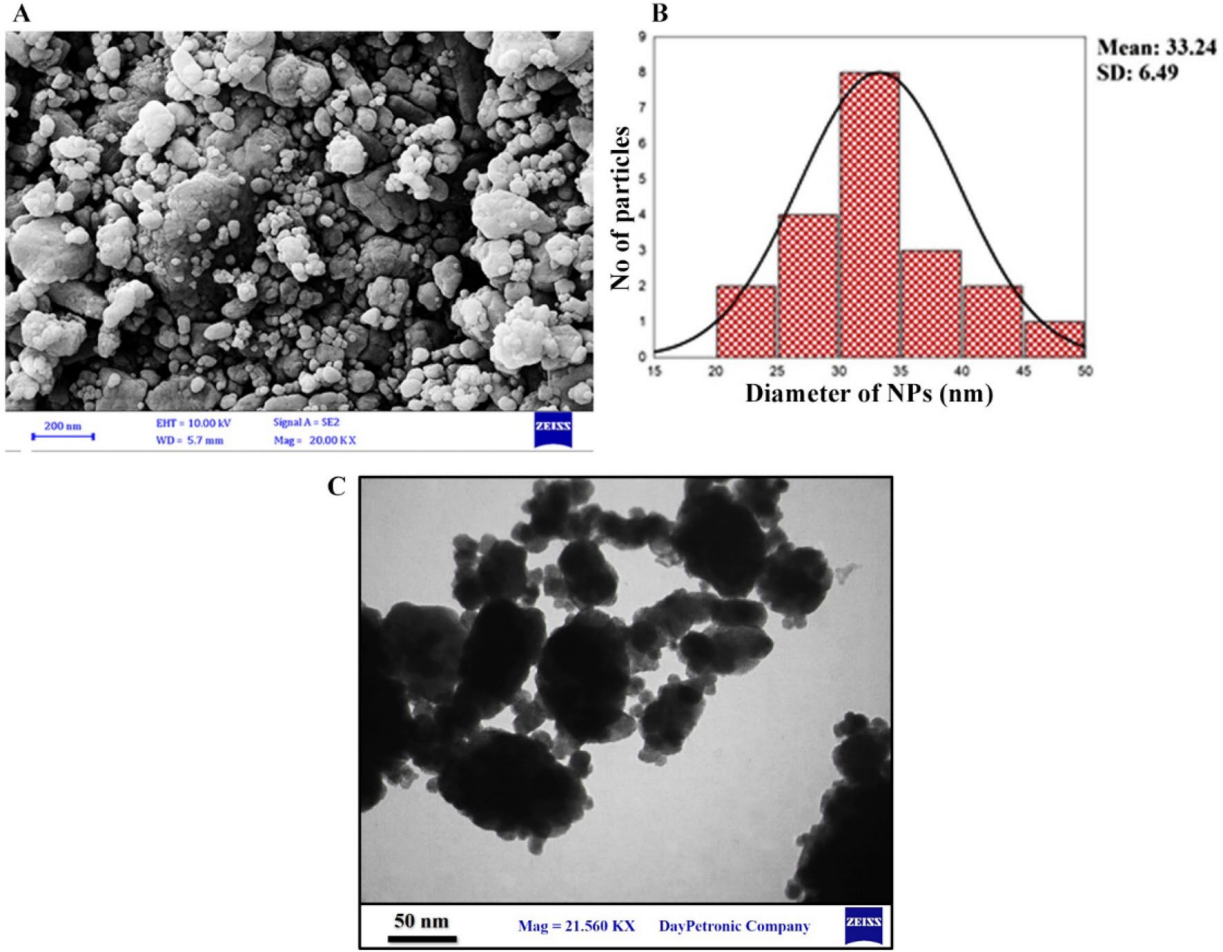
Structural and Morphological Properties of CuO NPs. (**A**) SEM image. (**B**) Diameter (nm) of CuONPs ranged between 33.24 ± 6.49 nm. (**C**) TEM image.

SEM imaging outcomes indicated that prepared nanoparticles’ diameter ranged between 33.24 ± 6.49 nm (Fig. 2A,B). Dispersed spherical nanoparticles were evaluated for Brownian movement using DLS assay, which helped understand the correlation between NPs’ hydrodynamic length characteristics inside the dispersing medium. Mathematical calculations of light scattering intensity dynamics are vital to understanding the correlation between NPs’ hydrodynamic length. As depicted in Fig. 2C, the prepared CuONPs had a 16–31 nm diameter range.

### CuONPs increase in lactate dehydrogenase release

**Figure 3:**
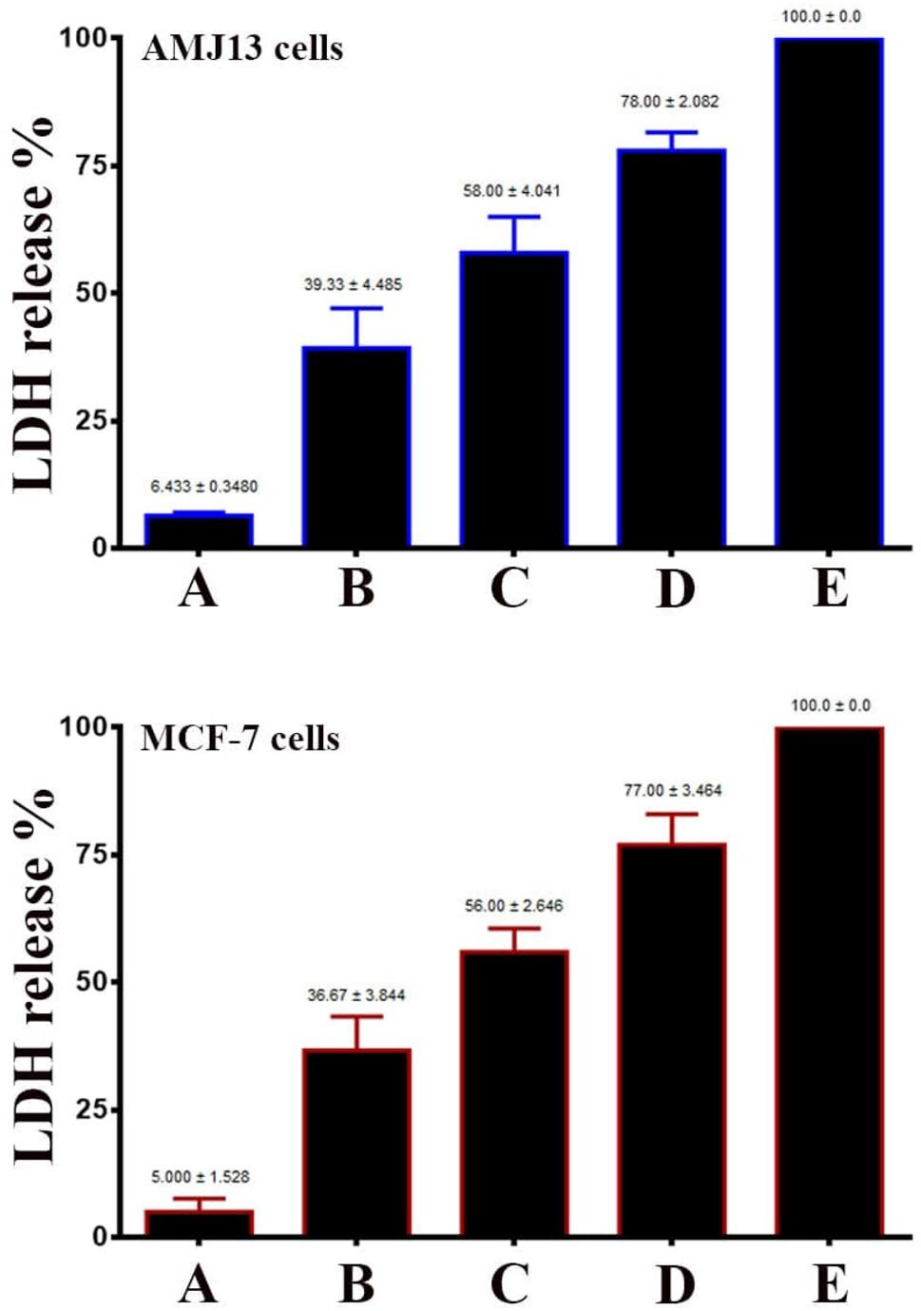
CuO NPs increase the release of lactate dehydrogenase (LDH) production. (**A**) control untreated cells. (**B**) Cells treated with 25 µg cells mL^−1^ of CuO NPs. (**C**) Cells treated with 50 µg cells mL^−1^ of CuO NPs. (**D**) Cells treated with 100 µg cells mL^−1^ of CuO NPs. 1% Triton-x was employed for positive control reference (**E**). LDH release was measured at 490 nm. Results are represented as mean ± SD.

The lactate dehydrogenase enzyme regulates the lactate-pyruvate transformation, which is critical for cellular energy production. Cell membrane integrity reduces when specific treated cells are introduced to this enzyme in the culture medium. LDH evaluation was used to determine the cytotoxic aspects of CuONPs on processed cells. Cell damage is followed by the cytoplasmic release of lactate dehydrogenase, triggering the conversion of the tetrazolium salt into formazan. Formazan formation is assessed using 490 nm, indicating the fraction and count of injured, affected, or dying cells. The gathered information could indicate CuONPs’ ability to enter the treated cells, trigger vesicle development, and enter them. Figure 3 indicates CuONPs’ capability to enhance LDH release, which is time-specific. CuO nanoparticles can penetrate cells and other biological components, leading to significant cellular degradation and triggering LDH production. At the same time, cells might consume 100–200 nm nanoparticles, triggering toxic phenomena like genetic changes and DNA damage.

Nanoparticle toxicity might be associated with processes that increase oxidative stress by interfering with the antioxidant system^31^. Free radicals such as ROS damage several membranes like those protecting the cell and mitochondria. Consequently, cellular constituents such as proteins, fatty acids, lipids, and nucleic acids, cause cellular death, reducing the proper functioning of the electronic transport mechanism. AMJ-13 and MCF-7 cells’ cytotoxicity might be associated with oxidative-stress caused cellular degradation. Moreover, time-based LDH production triggered by CuONPs exposure might be associated with the cell membrane. Along the same lines, time-based LDH production increase triggered by CuONPs might be associated with cellular membrane destruction, leading to the leakage of cellular enzymes like lactate dehydrogenase. Cytotoxicity initiation observed in this study aligns with the conclusions^32^.

### Anti-proliferative activity of CuONPs

We evaluated the cytotoxic aspects of the synthesized CuONPs on HBL-100, AMJ-13, and MCF-7. The CuONPs’ antitumor characteristics were assessed by evaluating the degree of proliferation inhibition concerning malignant cells. The outcomes indicated extremely toxic significant effects exerted by the synthesized CuONPs on AMJ-13 and MCF-7 cells.

**Figure 4:**
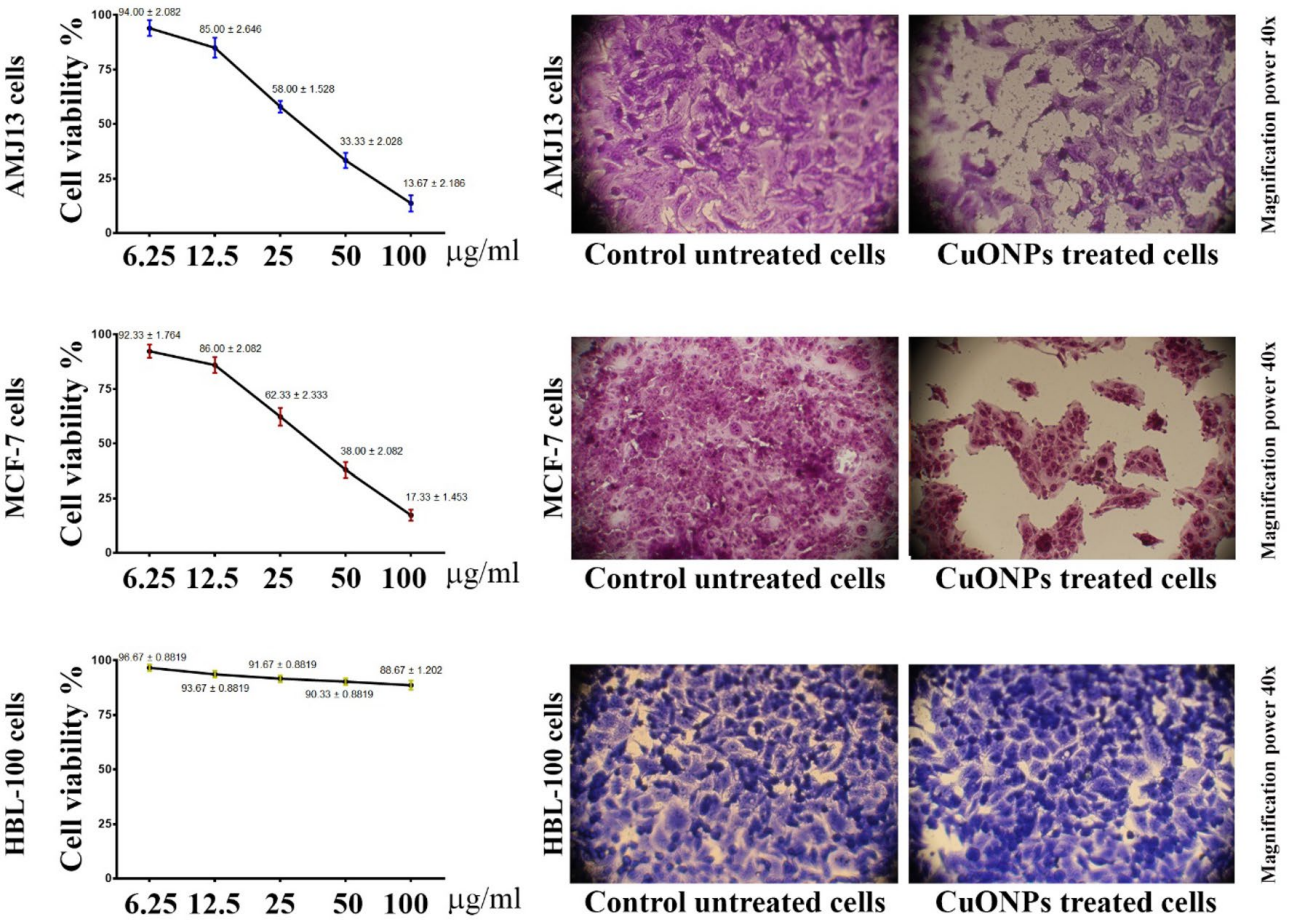
The Antiproliferative activity of CuO NPs against cancer cell lines. Triplicate samples of AMJ-13, MCF-7, and HBL-100 cells at a concentration of 1 × 10^4^ cells mL^−1^ were seeded. CuO NPs were added to these samples and incubation periods for 72 h. Cells were stained with MTT. The 492 nm wavelength was selected to assess absorbance.

While the outcomes did not indicate CuONPs induced cytotoxicity for the healthy cells (HBL-100), anticancer phenomena were detected by evaluating nanoparticles’ capability to reduce or block cancer cells, as depicted in Fig. 4. Hence, CuONPs can reduce cancer cell growth. AMJ-13 and MCF-7 lines were similarly impacted due to CuONPs exposure. The outcomes indicate the capability of CuONPs to reduce the proliferation of cancer cell lines. A 72-h CuONPs treatment provided to cancer cells significantly reduced proliferation capability, which aligns well with previous study outcomes^33^. In a study by Naik and Sellappan^34^. it was discovered that two A. muricata extracts showed higher genotoxic potential on breast cancer (MCF-7) cells when assessed using the alkaline comet assay. In another study, the ability of methanol extracts of Annona muricata leaves to induce apoptosis and/or stop the cell cycle was investigated in an effort to discover a potential strategy for controlling or inhibiting the growth of MCF-7 breast cancer^35^. CuO NPs demonstrated a wide range of cytotoxicity in our MTT cytotoxicity screening assay, with the majority of activity on AMJ-13 and MCF-7 cells but not on normal HBL-100 cells. The difficulty of anticancer medications to accurately differentiate cancer cells is one of the major problems facing cancer chemotherapy. Although CuO's cancer-specific toxicity is still unclear, CuO NPs' ability to selectively destroy cancer cells has clinical significance^36^. Recently, selective cytotoxicity of other nano-particles including gold nanoparticles (Au NPs) and silver nanoparticles (Ag NPs) on cancerous cells has been reported. Khorrami et al. observed that Ag NPs nanoparticles with an average size of 31.4 nm exhibited a preferential ability to kill MCF-7 cancerous cells as compared to normal L-929 fibroblast cells^37^. Vetten et al. indicated that AuNPs with an average size of 20 nm were considerably more toxic to Chinese hamster ovary cells in comparison to the same nanoparticles with the size of 14 nm^38^. Current findings in cancer research imply that the production of reactive oxygen species (ROS) through oxidative stress is a major method shared by multiple apoptotic stimuli^19^. ROS has been identified as an important signaling molecule for the start and completion of apoptosis^19^. Oxidative stress has shown the toxicity of CuO NPs in many human cell lines, including human lung epithelial (A549), human heart microvascular endothelial (HCMEC)^39^, and human hepatocarcinoma cell line (HepG2)^40^. CuO NPs' cytotoxicity towards mammalian cells varies depending on the kind of cell.

**Figure 5:**
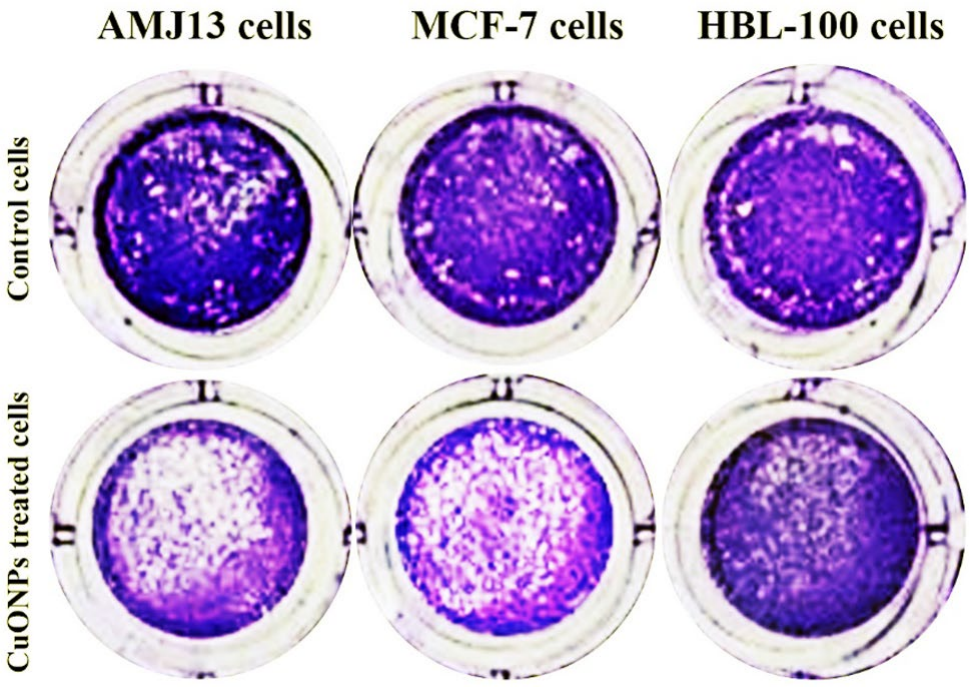
CuONPs inhibit the Colony-forming of MCF-7 and AMJ-13. A 24-h CuONPs treatment was provided at IC50 concentration, followed by crystal violet staining for 20 min.

Tumor cell colony development reduced after sustained CuONPs exposure for 24 h using (IC_50_) concentration, indicating that the cells were destroyed (Fig. 5). Hence, there was nanoparticle uptake at the cellular level, triggering apoptosis. Hence, the outcomes indicate that CuONPs are capable of triggering cell death. These results demonstrate that CuONPs selectivity reduces AMJ-13 and MCF-7 cell proliferation. The biomolecules in the plant extract function as efficient capping agents, hence facilitating the synthesis of NPs. NPs appear to be stabilized by a diverse range of mechanisms, notably electrostatic stability, steric stability, hydration force stability, and van der Waals forces. The stability of nanoparticles is crucial for their functions and applications^41^. Different processes, including apoptosis and necrosis^42^, can be utilized by NPs to inhibit cell viability. The induction of tumor cell apoptosis is an essential mechanism for anti-cancer agents^43,44^. The process of apoptosis, marked by morphological and biochemical changes, and the apoptosis of distinct cells in the same tissue do not occur simultaneously. Copper oxide nanoparticles demonstrated a concentration-dependent cytotoxic impact against breast cancer cells in our investigation.

### CuoNPs triggered apoptosis in breast cancer cells

Apoptosis is a vital homeostasis phenomenon that regulates different cell category concentrations^45^. The working hypothesis is that the synthesized CuONPs trigger apoptosis for processed AMJ-13, and MCF-7, indicating anti-proliferative characteristics. We evaluated if the nanoparticles triggered anti-proliferative activity concerning AMJ-13 and MCF-7 breast cancer cell lines and normal (HBL-100) lines for human cells concerning apoptosis triggers. The cells were treated using CuONPs at (IC_50_) concentration. The Ao/EtBr dual stain comprises fluorescent compounds mixed for identifying morphological transformation concerning the nucleus, providing distinct fluorescence.

**Figure 6:**
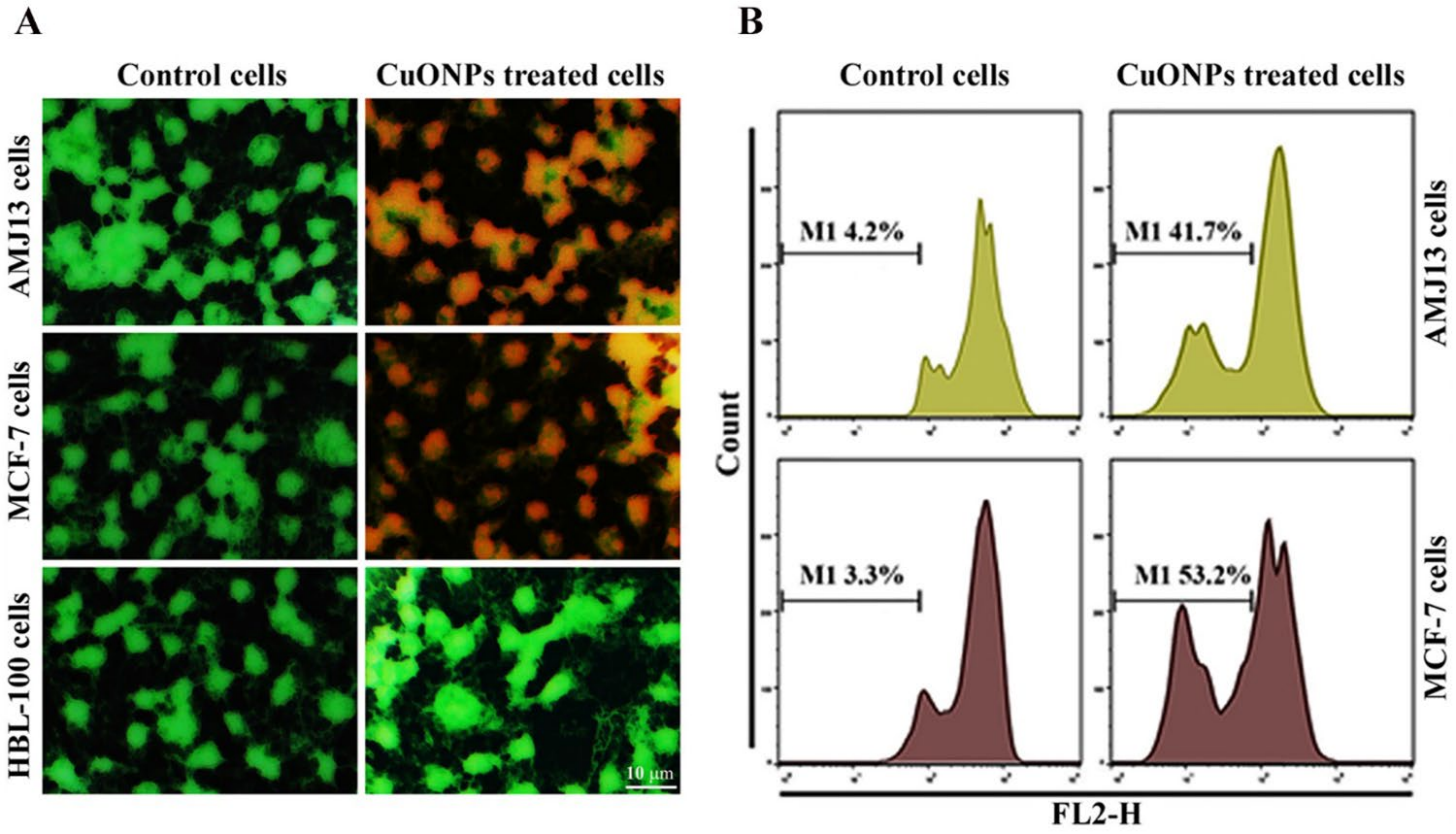
Apoptosis markers in cancer cells after treatment with CuO NPs. (**A**) Apoptotic indicators for AMJ-13, MCF-7, and HBL-100 cells exposed to CuONPs at IC_50_ concentrations for 24 h and stained for 2 min using AO/EtBr. Unexposed cells have an intact structure. Nevertheless, CuONPs exposure correlated with apoptotic aspects indicated using red stains. Scale bar 10 µm. (**B**) Cells cycle phase. Sub-G_1_ phase concerning MCF-7 and AMJ-13 cells’ flow cytometry.

The AMJ-13 and MCF-7 cells were exposed to CuONPs at IC_50_ levels for 24 h, followed by dual staining and visual assessment using fluorescence microscopy to ascertain nuclear structure change, as depicted in Fig. 6A. Therefore, tumor cells were exposed to CuONPs, indicating damage to the cell membrane and lysosome vacuoles compared to unexposed lines. No changes were identified for healthy cells. The outcomes demonstrated that CuONPs possessed potent cell-death triggering potential, which might be due to the significant membrane breaching ability. Ethidium bromide and acridine orange dyes were mixed to comprehensively assess the degree to which CuONPs could trigger the death of tumor cells. The outcomes reflected a bright green tinge, suggesting an undamaged nucleus. Nanoparticle-treated tumor cells had reduced cell membrane integrity compared to the control samples. Orange and red colors are characteristic of apoptotic cells. The present study also comprised experiments to assess the probable apoptosis-inducing ability of CuO NPs.

The constituent DNA was evaluated concerning the sub-G1 phase through flow cytometry; subsequently, PI was used to stain the DNA of processed AMJ-13 and MCF-7 cells. The sub-G1 phase outcomes suggested that the fraction of CuONPs-processed tumor cells rose to 41.7% from 4.2%, while MCF-7 cells increased to 53.2% from 3.3% (Fig. 6B). Study outcomes indicated that CuONPs demonstrated anti-proliferation characteristics by triggering cell death through apoptosis.

### CuONPs triggered upregulation of caspase-3 and caspase-9 expressions

**Figure 7:**
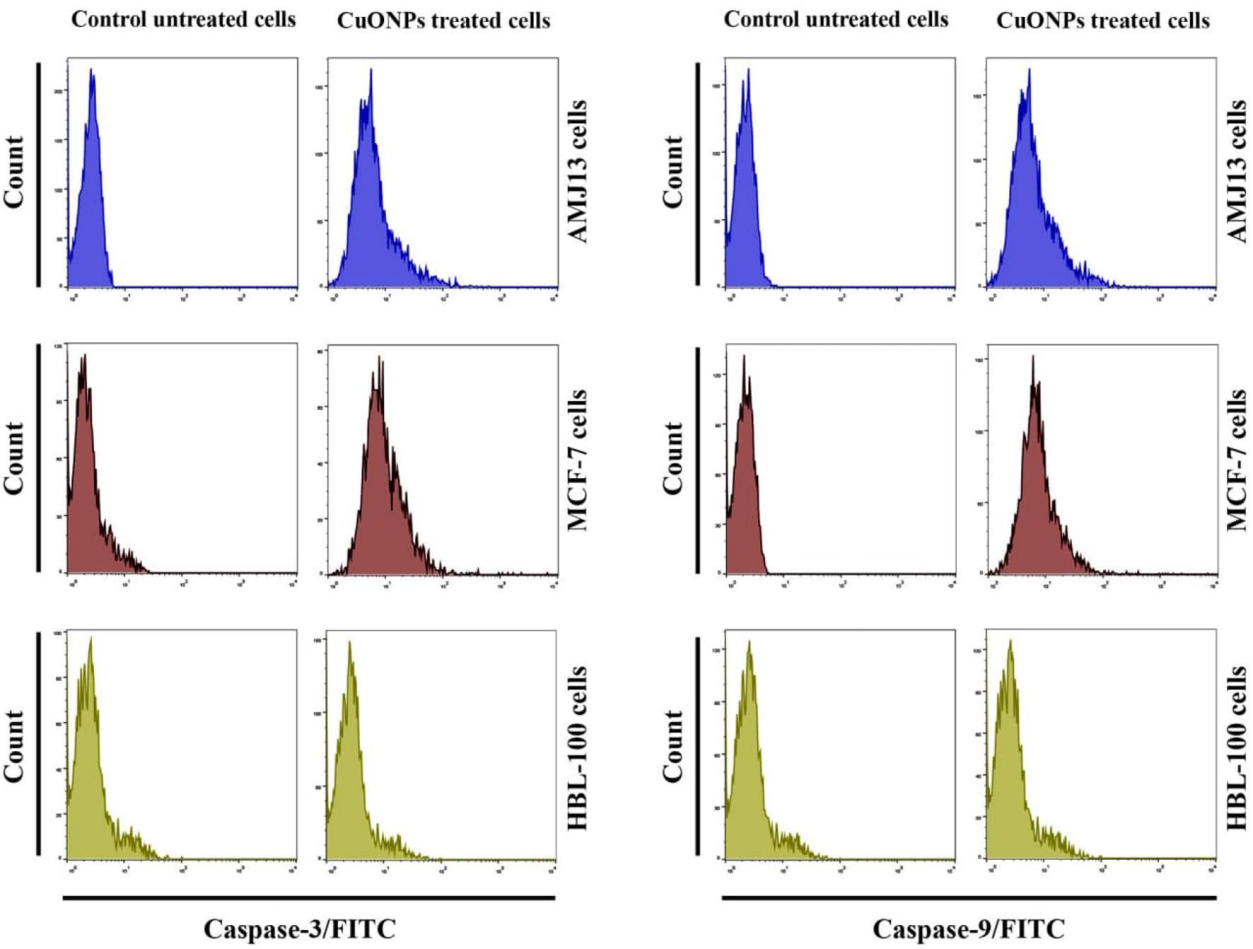
CuONPs up-regulated Caspase-3 and Caspase-9 expresion. Flow cytometry was used to analyse fluorescence histograms of immunolabeled caspase-3 and caspase-9.

The previously-tested assay suggests that CuONPs can trigger mitochondria-specific apoptosis, which requires two primary techniques: intrinsic (mitochondria-level regulation) and extrinsic (death receptor-mediated). The extrinsic pathway comprises molecule surface signals (Fas/FasL), causing the inclusion of the Fas-associated death domain (FADD)^46^. Caspases work in alignment with apoptosis triggers. The two major apoptosis pathways comprise the caspase-9 mediated mitochondrial and caspase-8 triggered death receptor pathways. Subsequently, executioner caspases -3 and -7 are triggered. The observations indicated significantly higher Caspase-3 and Caspase-9 levels (Fig. 7).

## Materials and methods

### Preparation of extracts

All the plant experiments were in compliance with relevant institutional, national, and international guidelines and legislation. The extraction protocol was performed according to Hemalatha et al.^47^ After the fruits were collected, the outer epicarp was peeled off, their seedless endocarp was shade dried, and 50 g of the dried endocarp was macerated into a powder. The fruit powder was combined with hot, boiling, distilled water, and kept in a shaker at 200 rpm for 4 h at 37 °C to create a 5% (w/v) suspension. The suspension was next cooled to room temperature before being passed through four layers of No. 1 Whatman filter paper and a 0.22 m filter (Millipore, Sigma). After being filtered, the aqueous extract was freeze-dried, and the powder was kept at − 20 °C until use.

### Cells and reagents

AMJ-13 (Iraqi patient's breast cancer cell line), MCF7, is an epithelial cell line isolated from the breast tissue of a patient with metastatic adenocarcinoma, and The HBL-100 cell line is an epithelial cell line derived in vitro from the milk of an apparently healthy woman. All cell lines were provided by the Iraqi Centre for Cancer and Medical Genetic Research (ICCMGR), Al-Mustansiriyah University, Baghdad, Iraq, which supplied the cells for the experiment. Trypsin–EDTA, fetal bovine serum, dimethyl sulfoxide (DMSO), RPMI-1640, 3-(4,5-Dimethylthiazol-2-yl)-2,5-diphenyltetrazolium bromide (MTT), and Triton X-100 were obtained from MO, USA. Kirkegaard & Perry Laboratories, Inc. (KPL) (Gaithersburg, MD, USA), while the other substances and reagents were analytical grade.

### Culturing of cell lines

All cell lines were cultured in PRMI-1640 (Sigma, USA) media, which contained 10% fetal bovine serum (FBS), 100 units/mL of penicillin, and 100 g/mL of streptomycin (Capricorn Scientific GmbH, Ebsdorfergrund, Germany). The cell lines were first cultivated as adherent monolayers grown at 37 °C in an incubator with 5% CO2 and a humidity atmosphere trypsinization for a short time using trypsin–EDTA (Capricorn Scientific GmbH, Ebsdorfergrund, Germany). These cells are routinely tested and verified.

### Collection and phytochemical extraction of *A. muricata*

In this study, the plant *Annona muricata* used in this study is not a globally threatened species. It was prepared by the department of biotechnology/ university of Technology from the local market in the city of Baghdad/Iraq. The phytochemical extraction was performed using 2 L chilled disinfected deionized distilled water mixed with 500 g *A. muricata* peel. To facilitate phytochemical removal uniformity, the sample was subjected to periodic vortexing during the 120-h standing period. Whatman filters (size 1) were used for staining the aqueous solution using a vacuum pump. A 4000-efficient Heldolph Laborata rotary evaporation apparatus was used for vacuum-based concentration for 12 h at 40 °C. Evaporation apparatus was used to dry the concentrates, and a powder form was obtained by treating the samples in hot air ovens set at 40 °C. The dry powder specimens obtained from the peel extracts were preserved at 4 °C using dark containers until used.

### Green synthesis of copper oxide nanoparticles mediated *A. muricata* peel

A conical flask was filled with 98 ml aqueous solution of 120.8 mg Copper II Nitrate mixed with 1000 mL deionised distilled water (0.5 mM). 2 ml *Annona muricata* extract was added for copper nanoparticle biosynthesis. Aluminium foil was used to seal the conical flasks stored in dark chambers at room temperature (25–26 °C) for 24 h. Subsequently, all liquids were removed at 120 °C using a hot air oven. The burnt brown content was removed from the beakers; a spatula was used to fill Eppendorf tubes with 1.5 ml solution, stored between 25 and 30 °C.

### Copper nanoparticle characterisation

#### UV–Vis spectroscopy

A Jenway 6715 UV–Vis spectrophotometer was employed for spectrophotometric assessment. Setups one and two were used to obtain 1 ml specimens after 8 and 24 h, respectively, and stored in separate cuvettes. The spectrophotometer was set at 1 nm resolution for the 200–700 nm range for assessing the prepared samples in order to determine the optical characteristics of copper nanoparticles.

#### Scanning electron microscopy SEM and TEM

The Carl Zeiss Sigma equipment was used for field emission scanning electron microscopy (FESEM) at 5.0 kV to determine the microstructure, and particle size characteristics of the CuONPs produced using the green approach. A carbon ribbon was taken, and 1 mg of copper nanoparticles were laid as a thin film coated using carbon, and several magnifications were used to capture the images. ImageJ software was employed to assess nanoparticle size distribution based on FESEM images.

### Lactate dehydrogenase release assay

Manufacturer recommendations were followed to test the lactate dehydrogenase (LDH) producing activity specimen. CuONPs were used to treat the cells for 24-, 48- and 72-h periods. The processed cells’ supernatant was moved onto a 96-well plate to assess LDH release characteristics by determining optical density at 490 nm^48^.

### MTT assay

A previously study was used to conduct the cytotoxicity test^49^. RPMI-provided microtiter plates were used for cell seeding using 1 × 10^5^ cells mL^−1^ concentration. The plates were adhered overnight; subsequently, several CuONPs concentrations were used. The triplicate samples were incubated for 72-h periods. MTT solution was then used to treat cells incubated for a predetermined period. Medium aspiration and DMSO addition were performed to assess absorbance (492 nm) using a microplate instrument. Regression assessment was employed to determine the concentration-specific to 50% cell growth inhibition (IC_50_)^50,51^.

### Clonogenicity assay

The experiment comprised 24-well plates to create AMJ-13, HBL-100, and MCF-7 cell cultures (10^5^ cells mL^−1^ for 24 h and subsequent CuONPs treatment). Once monolayer confluence was achieved, the cells were isolated from the medium, followed by PBS rinsing. Crystal violet (Sigma-Aldrich, MO, USA) was used to stain fixed cells, then dye removal by washing, and photography^52^.

### Acridine orange–ethidium bromide staining

Cells treated with copper nanoparticles were assessed for apoptosis induction using AO/EtBr staining (Sigma-Aldrich, USA). 12-well plates were used for cell seeding for 24 h; subsequently, copper nanoparticle treatment was used. PBS was used for two cell washing cycles. Solutions were prepared comprising identical cell volume and dual fluorescent dyes (10 µL) to assess the specimens using fluorescence microscopy^52,53^.

### Flow cytometry assay for apoptotic cells

A cell cycle phase identification kit was used to identify sub-G1 cells. HBL-100, AMJ-13, and MCF-7 cell plating were conducted at 1 × 10^6^ cell mL^−1^ concentration. CuONPs treatment was provided to the cells for 24 h, followed by centrifugation, PBS washing, fixing, and resuspension using propidium iodide (PI) and RNase A based staining buffer. The sub-G1 peak was identified using the flow cytometer’s FL2 channel^53^.

### Caspase-3 and caspase-9 level determination

Caspase-3 and caspase-9 activation states were assessed using fluorescent staining kits (Thermo Fisher Scientific, USA). The experiment comprised a 5 ml medium used to culture 4 × 10^4^ cells mL^−1^ SKOV-3 cancer cells, incubated at 37 °C for 24 h. Subsequently, the incubated specimens were treated with every compound for 24 h using 10 µg mL^−1^ concentration. Post-incubation, cells were removed, and 1 × ice-cold PBS was used to wash the samples twice. The pellets were gathered, and the growth medium was used to change cell density to 1 × 10^6^ cells mL^−1^. Subsequently, 1 mL of FITC-IETD-FMK was used to treat the cultivated cells, which were incubated for 60 min at 37 °C, to pinpoint caspase-9 and caspase-3. After incubation, a wash buffer (0.5 mL) was used twice to clean the cell samples. The stained samples were placed inside flow cytometry tubes to start the cytometry process. BD Accuri C6 software was used to assess the gathered data.

### Statistical analysis

GraphPad Prism 5 was used for statistical data processing for the unpaired *t* test^54^. Test results are indicated as the mean ± standard error corresponding to the assay-specific three replicate mean value^55^.

## Conclusions

Plants and the bioactive elements found in them have drawn a lot of interest in the development and discovery of novel non-toxic, environmentally friendly methods for the synthesis of metal nanoparticles because of their superior reducing ability, anticancer properties, and physiochemical characteristics of green CuONP synthesis. The additional benefit of this technology, which outperforms conventional chemical procedures, is that it prolongs the life of NPs. The present study conducted CuONPs synthesis using green techniques. Subsequently, the nanoparticles were assessed for anticancer characteristics concerning AMJ-13, MCF-7, and HBL-100 lines to ascertain the degree of cell proliferation and apoptosis. The outcomes indicate that CuONPs demonstrated strong anti-proliferative and pro-apoptotic effects on AMJ-13 and MCF-7 cell lines. Moreover, CuONPs treatment enhanced LDH production, attributed to cell membrane destruction, causing leakage of cellular enzymes. Hence, the study suggests that the synthesised CuONPs displayed anti-proliferative behaviour that triggered cell death through apoptosis.

## Data Availability

The datasets used and/or analysed during the current study available from the corresponding author on reasonable request.
